# Diamond Nanoparticles Modify Curcumin Activity: *In Vitro* Studies on Cancer and Normal Cells and *In Ovo* Studies on Chicken Embryo Model

**DOI:** 10.1371/journal.pone.0164637

**Published:** 2016-10-13

**Authors:** Barbara Strojny, Marta Grodzik, Ewa Sawosz, Anna Winnicka, Natalia Kurantowicz, Sławomir Jaworski, Marta Kutwin, Kaja Urbańska, Anna Hotowy, Mateusz Wierzbicki, André Chwalibog

**Affiliations:** 1 Division of Nanobiotechnology, Faculty of Animal Sciences, Warsaw University of Life Sciences, 8 Ciszewskiego Str., 02–786, Warsaw, Poland; 2 Division of Histology and Embryology, Faculty of Veterinary Medicine, Warsaw University of Life Sciences, 159 Nowoursynowska Str., 02–786, Warsaw, Poland; 3 Department of Pathology and Veterinary Diagnostics, Faculty of Veterinary Medicine, Warsaw University of Life Sciences, 159 Nowoursynowska Str., 02–786, Warsaw, Poland; 4 Division of Nano-nutrition, Faculty of Health and Medical Sciences, University of Copenhagen, Groennegaardsvej 3, 1870, Frederiksberg, Denmark; Laboratoire de Biologie du Développement de Villefranche-sur-Mer, FRANCE

## Abstract

Curcumin has been studied broadly for its wide range of biological activities, including anticancer properties. The major problem with curcumin is its poor bioavailability, which can be improved by the addition of carriers, such as diamond nanoparticles (DN). They are carbon allotropes, and are therefore biocompatible and easily taken up by cells. DN are non-toxic and have antiangiogenic properties with potential applications in cancer therapy. Their large surface makes them promising compounds in a drug delivery system for bioactive agents, as DN create bio-complexes in a fast and simple process of self-organisation. We investigated the cytotoxicity of such bio-complexes against liver cancer cells and normal fibroblasts, revealing that conjugation of curcumin with DN significantly improves its activity. The experiment performed in a chicken embryo model demonstrated that neither curcumin nor DN nor bio-complexes affect embryo development, even though DN can form deposits in tissues. Preliminary results confirmed the applicability of DN as an efficient carrier of curcumin, which improves its performance against cancer cells *in vitro*, yet is not toxic to an organism, which makes the bio-complex a promising anticancer agent.

## Introduction

Curcuminoids are primary compounds of turmeric, a broadly known spice that is mostly used in India and the surrounding regions. Curcuminoids are derived from the perennial herb *Curcuma longa*, obtaining their intense orange-yellow colour from its rhizomes [1]. Curcumin (Cur) [1,7-bis-(4-hydroxy-3-methoxyphenyl)-1,6-heptadiene-3,5-dione], is a water-insoluble diferuloylmethane (low molecular weight polyphenol) that was first isolated in 1815 by Vogel [2] and chemically described in 1910 by Lampe and Milobedzka. It is regarded as the most active compound of all turmeric fractions of curcuminoids [3,4].

There are hundreds of articles describing the biological activity of Cur and the number is still growing. It is currently known that Cur interacts with numerous molecular targets, such as transcription factors (e.g., NFκB, PPARγ, STAT family members, Notch), growth factors (e.g., HGF, PDGF, VEGF) and their receptors, cytokines (e.g., many interleukins, MIPa, TNFα), enzymes (e.g., FASN, COX-2, 5-LOX, MMP family members) and genes regulating cell cycle and apoptosis (e.g. Bax, beclin family members, caspases) [5]. The uses of Cur include the treatment of various inflammatory diseases, rheumatism, swellings and abdominal problems as well as wound healing [4]. Cur was also proved to have excellent antioxidant capacity, what was demonstrated by chemical tests, as well as by *in vitro* assays performed on chicken red blood cells [6]. Since the 1990s, interest in Cur has been focused on its potential as an anticancer agent [7,8]. The major problem with Cur is its low bioavailability, which is due to its highly hydrophobic character, poor absorption from the gut, limited distribution within body tissues, low intrinsic activity and rapid excretion [2,9].

The inclusion of carriers can improve the bioavailability of Cur; hence, various Cur delivery systems have been investigated, including liposomes, biodegradable polymers, dendrimers and cellulose nanoparticles [10,11]. The application of nanotechnology for Cur usage has improved its chemical stability, cellular uptake and antioxidant effects, prolonged blood circulation and increased its anti-inflammatory and antitumor effects. Nanoparticles of diamond (DN), also called nanodiamond, are one of the carbon allotropes; therefore, they are non-toxic, easily taken up by cells and have a high biocompatibility [12–15]. Furthermore, DN have a large surface area and a high absorption capacity [16]. DN can create bio-complexes with organic molecules such as L-glutamine [17] in a fast and simple process, called self-organisation, which makes DN a sufficient carrier of bioactive agents. Moreover, DN were demonstrated to have antiangiogenic properties [18,19] and to inhibit tumour growth [20], as angiogenesis is one of the major factors promoting tumour development. These features promote DN as a component of drug-delivery systems for tumour treatment.

We hypothesised that Cur conjugated with DN could create a stable bio-complex, which would improve Cur bioavailability and activity against tumour cells, being non-toxic and highly biocompatible with an organism. In order to evaluate the cytotoxic effects of Cur, DN and their bio-complexes against cancer and normal cells, we performed a series of assays on the HepG2 liver cancer cell line and fibroblast primary cultures, including the evaluation of cell viability, membrane integrity, morphology and apoptosis. The second part of the experiment was performed on a chicken embryo to evaluate a potential toxicity of Cur, DN and their complexes in a model organism. We evaluated the development, body and organ weights, blood morphology and biochemistry as well as brain and liver histology.

## Materials and Methods

### Ethics statement

The experimental procedures were performed in accordance with Polish legal regulations concerning experiments on animals (Dz. U. 05.33.289). The experimental protocols were approved by the III Local Ethics Commission for Experimentation on Animals at Warsaw University of Life Sciences, Poland.

### Hydrocolloids of curcumin, diamond nanoparticles and their bio-complexes

Diamond nanoparticles, produced by the detonation method, purity >95%, were obtained from Skyspring Nanomaterials (Houston, TX, USA). Curcumin was obtained from LKT Laboratories Inc. (St. Paul, MN, USA).

Cur and DN were dissolved in ultra-high purity deionised water using sonication at a final concentration of 500 mg/L. Bio-complexes of DN and curcumin at a ratio of 3:1 (3Cur:1DN group) and a ratio of 1:3 (1Cur:3DN group) were obtained by the self-organisation process using sonication for 30 min in an ultrasonic bath (Sonorex Super RK 514H, Bandelin Electronic, Germany). For experiments performed on cells, bio-complexes 3Cur:1DN and 1Cur:3DN were diluted in culturing media to final concentrations 60 and 100 mg/L (which corresponds to 15Cur:45DN mg/L, 45Cur:15DN, 25Cur:75DN mg/L and 75Cur:25DN mg/L), whereas Cur and DN were diluted respective to their concentrations within bio-complexes (15, 25, 45 and 75 mg/L). Dilutions were prepared freshly before every test.

The shape and size of DN and bio-complexes were inspected by transmission electron microscopy (JEM-2000EX; JOEL Ltd., Tokyo, Japan) at 200 keV. Zeta potential measurements of hydrocolloids were carried out at 25°C by the laser dynamic scattering-electrophoretic method with a Smoluchowski approximation using a Zetasizer Nano-ZS90 (Malvern, Worcestershire, UK). Each measurement was repeated three times.

### *In vitro* cell culture experiment

#### Cell cultures

The human hepatocarcinoma HepG2 cell line was obtained from the American Type Culture Collection (ATCC). The human fibroblast primary cell culture was isolated from human skin by the Nalecz Institute of Biocybernetics and Biomedical Engineering PAS, Warsaw, Poland.

HepG2 cells were maintained in Dulbecco’s Modified Eagle's Medium (DMEM, Gibco™, Thermo Scientific, Waltham, MA, USA) and human fibroblasts were maintained in Medium 106 (Gibco™). Media were supplemented with 10% foetal bovine serum (FBS, Gibco™), penicillin (100 U/mL) and streptomycin (100 mg/mL) and cultures were maintained at 37°C in a 5% CO_2_ and humidified atmosphere.

#### Viability assay

Cells were seeded on 96-well microplates (Nest Scientific, Rahway, NJ, USA) at a density of 4 × 10^5^ (HepG2) and 3 × 10^5^ (human fibroblasts) in 100 μl of medium per well. The next day, the medium was removed and replaced with fresh medium containing dilutions of Cur and diamond nanoparticles (at concentrations of 15, 25, 45 and 75 mg/L) and their complexes at concentrations of 60 and 100 mg/L (Cur15:DN45, Cur45:DN15, Cur25:DN75 and Cur75:DN25). Cell viability was assessed after 24 h by MTT assay, where yellow soluble tetrazolium salt is converted to purple formazan crystals. MTT was dissolved in PBS (5 mg/ml) and 15 μl were added per well. After 3 h, solubilisation detergent (10% SDS, 0.01 M HCl) was added (100 μl /well). Spectrophotometer readings were performed on the following day at 570 nm on an Infinite® 200 PRO microplate reader with i-control™ Software (Tecan Group Ltd., Männedorf, Germany). USA). Cell viability was expressed as the percentage of the control group viability, which was 100%. Calculations were performed from the following formula: ABS_test_- ABS_blank_) /(ABS_control_-ABS_blank_), where “ABS_test_” is the absorbance of wells exposed to the treatment, “ABS_control_” is the absorbance of control wells, and “ABS_blank_” is the absorbance of wells without cells, with media containing the respective treatment for each well.

#### Cell membrane integrity assay

A lactate dehydrogenase (LDH) leakage test (LDH-based In Vitro Toxicology Assay Kit, Sigma-Aldrich) was used to evaluate cell membrane integrity. If the membrane is damaged, intracellular LDH molecules are released into the culture medium and tetrazolium dye from the assay is stoichiometrically converted. Reduced nicotinamide adenine dinucleotide (NADH+) is used for the detection of the converted dye.

HepG2 and human fibroblasts were seeded on 96-well plates and treated with Cur, DN and their complexes as described above for the viability assay.

After 24 h of incubation, 25 μl of the culture medium was transferred to a clean, 96-well microplate. A total of 50 μL of the lactate dehydrogenase assay mixture was added to each well. The plate was covered and incubated for 20 min at room temperature. The remaining cells with 75 μl of media were used for the calculation of cell number, which was performed by the MTT assay.

The absorbance for LDH measurements was recorded at 490 nm and background at 690 nm was subtracted. LDH leakage was as the percentage of the control group LDH release, which was 100%. Calculations were performed from the following formula: ABS_test_/ABS_control_, where “ABS_test_” is the absorbance of wells exposed to treatment, “ABS_control_” is the absorbance of the control wells. Results were then normalized to the number of cells calculated by the MTT assay by dividing the % of LDH leakage by the % of living cells for each well.

#### Cell death type

The type of death was evaluated by propidium iodide (PI) staining and annexin V (AnnV) assay (Thermo Scientific, Waltham, MA USA), where PI is a fluorescent dye incorporated into nucleic acids of dead and disrupted cells and annexin V conjugated to the fluorescent marker Alexa Fluor 488 binds specifically to phosphatidylserine, which is exposed on the external surface of the cell membrane during apoptosis. PI-positive cells are considered necrotic, PI/AnnV-positive cells as late apoptotic and AnnV-positive cells as apoptotic. Live cells remain unstained.

Cells were seeded on 12-well plates (Nest Scientific) at a density of 4 × 10^5^ (HepG2) and 5 × 10^4^ (human fibroblasts) in 1 ml per well. The next day, the medium was replaced with fresh medium containing dilutions of Cur and DN nanoparticles at concentrations of 15 and 45 mg/L, and their complexes at concentrations of 60 mg/L (Cur 15:DN45, Cur45:DN15). After 24 h, the medium was removed and the cells were washed with PBS. The staining procedure was performed according to the manufacturer's protocol. Readings were performed with BD FACSCalibur™ cytometer (Becton Dickinson, Franklin Lakes, NJ, USA). Eight thousand events were recorded for each sample. Plots were generated using Flowing Software 2.5.1 (Perttu Terho, Turku, Finland).

#### Cell morphology

Cells were seeded on 12-well plates (Nest Scientific) at a density of 4 × 10^5^ (HepG2) and 5 × 10^4^ (human fibroblasts) in 1 ml per well. The next day, the medium was replaced with fresh medium containing dilutions of Cur and DN nanoparticles at concentrations 50 mg/L, and their complexes at concentrations 100 mg/L (Cur25:DN75, Cur75:DN25). After 24 h of incubation (37°C, 5% CO_2_), the medium was removed and the cells were washed twice with PBS. One millilitre of May-Grünwald stain was added per well and after 3 min, it was diluted with an equal amount of PBS. After 3 min, the stain was replaced with 1 ml of Giemsa stain (diluted 1:20 in distilled water). After 30 min, the stain was removed and the cells were washed thoroughly with distilled water. The evaluation was performed under a microscope connected to a digital camera.

### *In ovo* chicken embryo experiment

#### Design of the experiment

The fertilized eggs (*Gallus gallus*, strain Hubbard) were supplied by a commercial, local hatchery (Marylka, Poland). Fertilized eggs (150) were divided into six groups as follows: without injection (Control), injected with phosphate-buffered saline (Placebo), injected with hydrocolloid of Cur (Cur group), injected with hydrocolloid of diamond nanoparticles (DN group) and injected with hydrocolloid of Cur with diamond nanoparticles at a ratio of 3:1 (3Cur:1 DN group) and at a ratio of 1:3 (1Cur:3DN group).

Embryonic day 0 (E0) was designated as the day when the eggs were placed into the incubator. The experimental solution was given *in ovo*, into the albumen on the E0 day of incubation, by injection of 0.3 ml of solution, using a sterile insulin syringe. After the injection, the holes were sealed with sterile tape and the eggs were then incubated for 20 days under standard conditions (temperature 37.8°C, humidity 55%, turned once per hour during the first 18 days, and from day 19 at a temperature of 37°C and humidity 60%).

At day 20 of incubation, embryo mortality was evaluated and the developmental status of chicken embryos was compared with the stages of development described by Hamburger and Hamilton [21]. The embryos and organs (liver, brain, spleen and heart) were weighed and blood samples were collected from neck blood vessels after decapitation.

#### Blood morphology

A droplet of each blood sample was used to prepare blood smears, stained by the Pappenheim method with May-Grünwald and Giemsa solutions (Sigma-Aldrich). Blood morphology was recorded under a light microscope (DM750; Leica Microsystems GmbH, Wetzlar, Germany) connected to a digital camera, using LAS EZ v 2.0 software (Leica). Total white blood cells were counted and shown as the % of all counted cells in blood samples.

#### Blood serum biochemistry

Blood samples were centrifuged (3000 rpm, 15 min; Thermo Fisher Scientific, Waltham, MA, USA) and activities of alanine aminotransferase (ALT) and asparagine aminotransferase (AST) were measured. ALT and AST were examined using the Vitros DT 60 II chemistry system (Johnson and Johnson, New Brunswick, NJ, USA).

#### Brain and liver histology

Sampled chicken brains and livers were fixed in 10% buffered formaldehyde, pH 7.2 (Sigma-Aldrich), then dehydrated in a graded series of ethanols, embedded in paraffin (Paraplast, Sigma-Aldrich), cut into sections (5 μm) with a microtome (Leica, Nussloch, Germany) and stained using haematoxylin (POCH S.A, Gliwice, Poland) and eosin (BDH Laboratory Supplies, Poole, Dorset, United Kingdom). Morphology was evaluated using a light microscope (DM750; Leica) connected to a digital camera and a computer analysis system (Leica).

#### Statistical analysis

The data are shown as a mean and standard error of the mean (SEM) or standard deviation (SD). Statistical analysis of data was carried out using one-factorial analysis of variance (ANOVA) with Duncan’s post-test to determine the differences between the groups for *in ovo* results or two-factorial ANOVA for *in vitro* results with Bonferroni’s post-tests, where DN and Cur were factors. Effects with *P*-values <0.05 were considered significant.

The analysis was performed using Statgraphics Centurion ver. XV software (Warrenton, VA, USA). Plots for cells viability and LDH leakage were created using GraphPad Prism 5.0 (La Jolla, CA, USA).

## Results

### Bio-complex analysis

**Fig 1:**
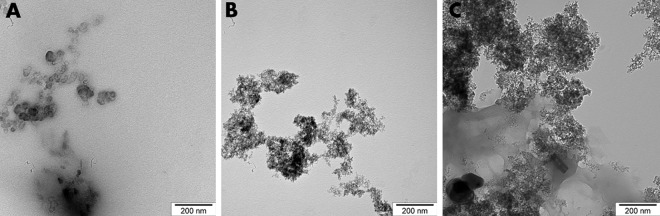
TEM examination of Cur (A), DN (B) and a bio-complex of Cur:DN in a ratio of 1:3 (C).

TEM examination confirmed the formation of complexes between Cur and DN. The average size of a single DN was from 2 to 5 nm (Fig 1A). The size of Cur particles was not defined because they created a cloud-shaped structure (Fig 1B). The size of bio-complexes of Cur and DN could not be precisely defined; nevertheless, it was estimated to be in the range of 10 to 100 nm (Fig 1C). The mean zeta potential values of Cur, DN and bio-complexes (3Cur:1DN and 1Cur:3DN) were negative, indicating the stability of colloids: -29.2 mV, -37.3 mV, -31.1 mV and -28.7 mV, respectively for Cur, DN, 3Cur:1DN and 1Cur:3DN-37.3 mV in DN solution to -28.3 mV in 1Cur:3DN solution.

### *In vitro* cell culture experiment

#### Cell viability

**Fig 2:**
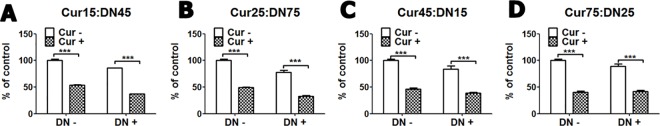
**HepG2 cell viability measured by mitochondrial activity depletion after DN, Cur and DN:Cur bio-complexes treatment, separately for each of the used bio-complexes the DN and Cur concentrations in each of the bio-complexes were 15, 25, 45 and 75, defining the concentrations in mg/L: A**–Cur15:DN45 (*P* < 0.0001 for Cur effect, *P* = < 0.0001 for DN effect, *P* = 0.4813 for Cur x DN effect); **B**–Cur25:DN75 (*P* < 0.0001 for Cur effect, *P* = 0.0001 for DN effect, *P* = 0.3422 for Cur x DN effect); **C–**Cur45:DN15 (*P* < 0.0001 for Cur effect, *P* = 0.0043 for DN effect, *P* = 0.1610 for Cur x DN effect); **D**–Cur75:DN25 (*P* < 0.0001 for Cur effect, *P* = 0.1207 for DN effect, *P* = 0.0722 for Cur x DN effect). Graphs show mean ± SD, *** *P* <0.001, for Cur effect by Bonferroni post-tests following two-way ANOVA with Cur and DN factors.

**Fig 3:**
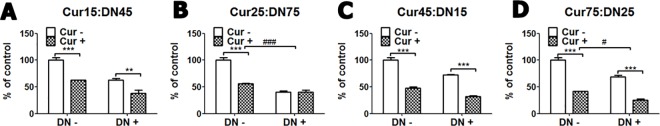
**Fibroblast cell viability measured by mitochondrial activity depletion after DN, Cur and DN:Cur bio-complexes treatment, separately for each of the used bio-complexes the DN and Cur concentrations in each of the bio-complex were 15, 25, 45 and 75, defining the concentrations in mg/L: A**–Cur15:DN45 (*P* < 0.0001 for Cur effect, *P* = < 0.0001 for DN effect, *P* = 0.1568 for Cur x DN effect); **B**–Cur25:DN75 (*P* < 0.0001 for Cur effect, *P* < 0.0001 for DN effect, *P* < 0.0001 for Cur x DN effect); **C–**Cur45:DN15 (*P* < 0.0001 for Cur effect, *P* = < 0.0001 for DN effect, *P* = 0.0615 for Cur x DN effect); **D**–Cur75:DN25 (*P* < 0.0001 for Cur effect, *P* = < 0.0001 for DN effect, *P* = 0.0495 for Cur x DN effect). Graphs show mean ± SD, ** *P* <0.01, *** *P* <0.001, for Cur effect by Bonferroni post-tests following two-way ANOVA with Cur and DN factors and #*P* <0.05, ###*P* <0.001 for the Cur x DN interaction.

Cur affected cell viability in both the HepG2 cell line and fibroblast cultures, which was demonstrated by decreased mitochondrial activity (Figs 2 and 3). Moreover, the linking of Cur to DN decreased cell viability in comparison with Cur alone in the same concentration as in bio-complexes. Among them, bio-complexes with the ratio 1Cur:3DN seemed to be the most effective and two-way ANOVA showed significant interaction between Cur and DN for this ratio in fibroblasts (Fig 3). DN alone impacted the viability of fibroblasts, but not that of HepG2; however, the effect on fibroblasts was less prominent than that of Cur as part of bio-complexes. The half maximal inhibitory concentration (IC_50_) of Cur was calculated to be between 25.5 and 31.9 mg/L for the HepG2 cell line and 46.8 mg/L for fibroblasts.

#### Cell membrane integrity

**Fig 4:**
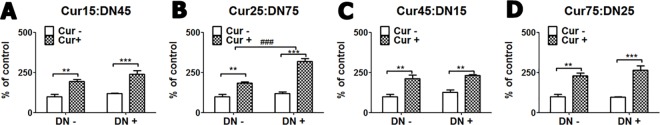
**HepG2 membrane integrity measured by LDH leakage after DN, Cur and DN:Cur bio-complexes treatment, separately for each of the used bio-complexs the DN and Cur concentrations in each of the bio-complexes were 15, 25, 45 and 75, defining the concentrations in mg/L: A**–Cur15:DN45 (*P* < 0.0001 for Cur effect, *P* = 0.0667 for DN effect, *P* = 0.4338 for Cur x DN effect); **B**–Cur25:DN75 (*P* < 0.0001 for Cur effect, *P* = 0.0004 for DN effect, *P* = 0.0024 for Cur x DN effect); **C–**Cur45:DN15 (*P* < 0.0001 for Cur effect, *P* = 0.1844 for DN effect, *P* = 0.8649 for Cur x DN effect); **D**–Cur75:DN25 (*P* < 0.0001 for Cur effect, *P* = 0.4403 for DN effect, *P* = 0.3246 for Cur x DN effect). Graphs show mean ± SD, ** *P* <0.01, *** *P* <0.001, for Cur effect by Bonferroni post-tests following two-way ANOVA with Cur and DN factors and ###*P* <0.001 for the Cur x DN interaction.

**Fig 5:**
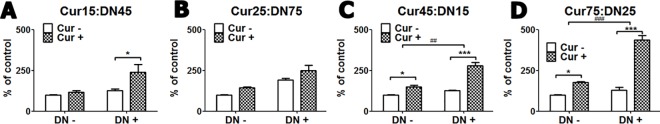
**Fibroblast membrane integrity measured by LDH leakage after DN, Cur and DN:Cur bio-complexes treatment, separately for each of the used bio-complex the DN and Cur concentrations in each of the bio-complexes were 15, 25, 45 and 75, defining the concentrations in mg/L: A**–Cur15:DN45 (*P* = 0.0336 for Cur effect, *P* = 0.0171 for DN effect, *P* = 0.0952 for Cur x DN effect); **B**–Cur25:DN75 (*P* = 0.0136 for Cur effect, *P* = 0.0003 for DN effect, *P* = 0.7073 for Cur x DN effect); **C–**Cur45:DN15 (*P* < 0.0001 for Cur effect, *P* = 0.0001 for DN effect, *P* = 0.0019 for Cur x DN effect); **D**–Cur75:DN25 (*P* < 0.0001 for Cur effect, *P* < 0.0001 for DN effect, *P* = 0.0001 for Cur x DN effect). Graphs show mean ± SD, * *P* <0.05, ** *P* <0.01, *** *P* <0.001, for Cur effect by Bonferroni post-tests following two-way ANOVA with Cur and DN factors and ##*P* <0.01, ###*P* <0.001 for the Cur x DN interaction.

Cur and Cur:DN bio-complexes affected cell membrane integrity in HepG2 cells, which led to significant leakage of LDH (Figs 4 and 5). For fibroblasts, the same effect was recorded only in cells treated with bio-complexes, confirming the interaction between DN and Cur. DN did not disrupt cell membranes, neither in HepG2 nor in fibroblasts, except at the concentration of 75 mg/L in fibroblasts, where the leakage was noticeable.

#### Cell death type

**Fig 6:**
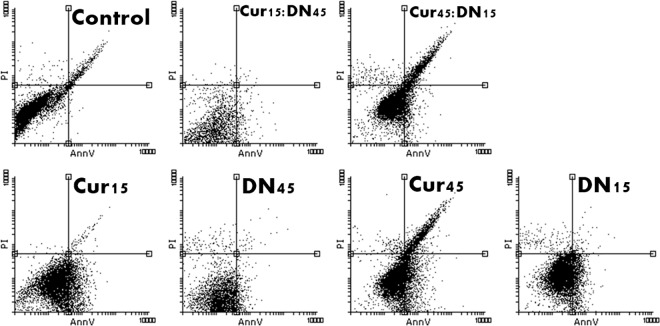
Dot blot/scatter diagrams of PI/AnnV staining of HepG2 cells treated with curcumin (Cur), diamond nanoparticles (DN) and their bio-complexes (Cur:DN) at concentrations of 15 and 45 mg/L. The transition of cells to AnnV quadrants visible for Cur and bio-complex-treated cells.

**Fig 7:**
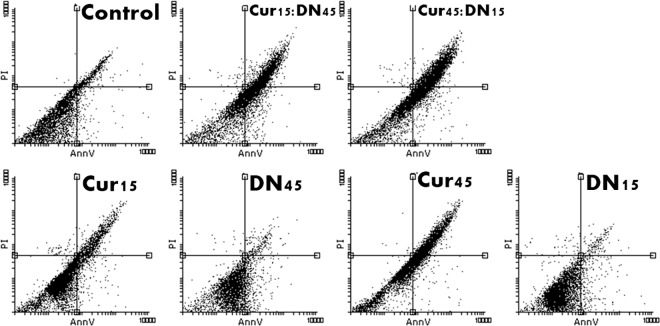
Dot blot/scatter diagrams of PI/AnnV staining of fibroblasts treated with curcumin (Cur), diamond nanoparticles (DN) and their bio-complexes (Cur:DN) at concentrations of 15 and 45 mg/L. The transition of cells to AnnV quadrants visible for bio-complex-treated cells.

The assay we used allowed us to detect one of the earliest stages of apoptosis, which is phosphatidylserine externalization. The % of necrotic cells in all samples was not significant in contrast to that of apoptotic cells (Figs 6 and 7). In both HepG2 cells and fibroblasts, Cur induced programmed cell death, alone and within bio-complexes (Table 1). The latter effect was especially enhanced in fibroblasts.

**Table 1:** Total % of apoptotic cells (both early and late apoptosis) in HepG2 cells and fibroblasts (Fib) treated with curcumin (Cur), diamond nanoparticles (DN) and their bio-complexes (Cur:DN) at concentrations of 15 and 45 mg/L.

	Control	Cur15	Cur45	DN15	DN45	Cur15:DN45	Cur45:DN15
**HepG2**	9.43	59.34	75.23	59.30	51.54	58.19	55.04
**Fib**	14.65	13.69	22.77	6.81	10.72	33.49	41.85

#### Cell morphology

**Fig 8:**
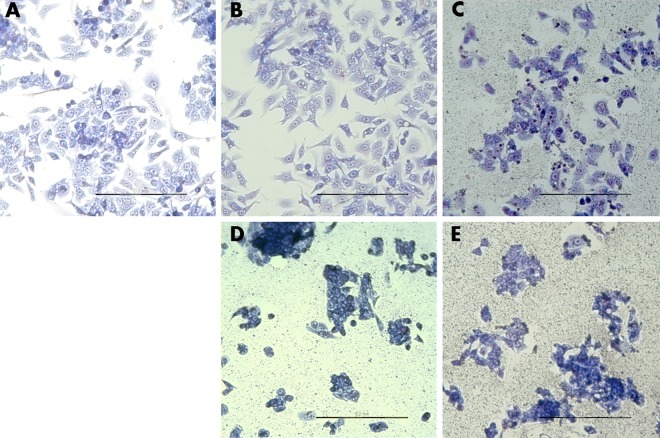
HepG2 cell morphology. **Control (A), Cur45 (B), DN45 (C), Cur75:DN25 (D), Cur25:DN75 (E).** DN visible on the surface of wells (C–E). Scale bar indicates 200 μm.

**Fig 9:**
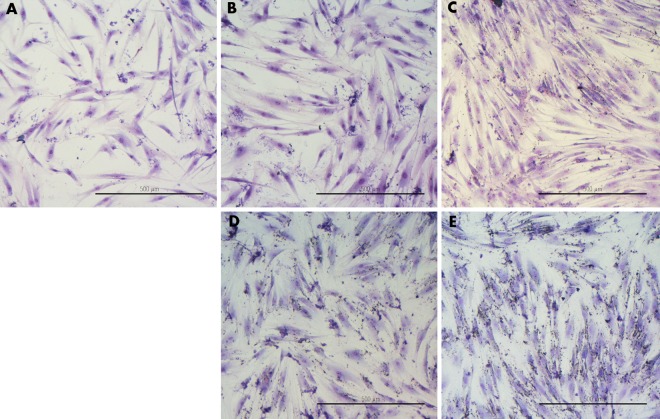
Fibroblast cell morphology. **Control (A), Cur45 (B), DN45 (C), Cur75:DN25 (D), Cur25:DN75 (E).** Scale bar indicates 500 μm.

Cell morphology confirmed the assay results: the number of cells treated with Cur:DN bio-complexes was diminished, which was clearly visible in the HepG2 line (Fig 8). Moreover, cells showed a tendency to agglomerate: they were shrunken and lost their shape. In wells where cells were treated with DN and Cur:DN, deposits of DN were visible across the whole well. Interestingly, fibroblasts treated with DN seemed to form more regular structures than control cells (Fig 9).

#### Mortality, growth and development of chicken embryos

The administration of Cur, DN and their bio-complexes slightly affected the mortality of embryos, which ranged from 8% in the Control and 3Cur:1DN groups to 15% in the Cur group (Table 2). In all groups, chicken embryos developed normally without abnormalities, in accordance with the Hamburger and Hamilton stages[21]. At day 20 of incubation (stages 44‒45), chickens were completely developed and yolk sacs were partly enclosed in the body cavity. Statistical analysis of embryo weight showed no significant difference between groups; similarly, the brain, liver and spleen weights were not significantly different. However, the weight of the heart was significantly lower in DN and Cur:DN groups than in the control group.

**Table 2:** The mortality, abnormalities and body and organ weights in 20-day-old chicken embryos after *in ovo* injection of curcumin (Cur), diamond nanoparticles (DN) and their bio-complexes (3Cur:1DN; 1Cur:3DN).

	Groups						ANOVA	
	Control	Placebo	Cur	DN	3Cur:1DN	1Cur:3DN	SEM	*P*-Value
**Mortality [%]**	8	10	15	9	8	11	-	-
Embryo body mass [g]*	48.6	46.6	49.2	47.9	46.9	47.9	1.25	NS
**Liver [g/100g body mass]**	2.321	2.115	2.225	2.254	2.145	2.587	0.0927	NS
**Brain [g/100g body mass]**	2.651	2.758	2.614	2.514	2.801	2.721	0.1175	NS
**Spleen [g/100g body mass]**	0.035	0.037	0.036	0.036	0.033	0.035	0.0021	NS
**Heart [g/100g body mass]**	0.768^a^	0.743^c^	0.732^c^	0.712^b^	0.723^b^	0.713^b^	0.0372	0.04

^abc^ values within rows with different superscripts are significantly different

NS statistically not significant

*Embryos weighed with the yolk sac

#### Blood morphology and biochemical indices

**Fig 10:**
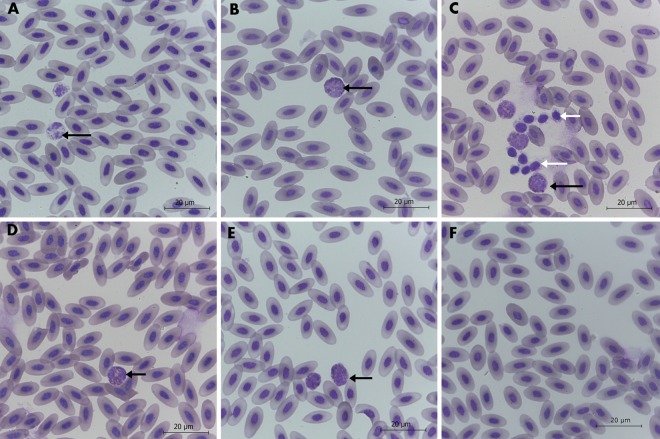
Images of stained chicken blood smears. **Groups: Control (A), Placebo (B), Cur (C), DN (D), 3Cur:1DN (E), 1Cur:3DN (F).** Heterophils (black arrows) and thrombocytes (white arrows). Scale bar indicates 20 μm.

The blood smears from the Control, Placebo and treated groups did not show symptoms of inflammation and blood cell deformation (Fig 10). However, in the Cur group, the number of white blood cells was significantly higher than in the other groups.

There was a tendency toward increased ALT and AST levels in the DN group compared with the other groups (Table 3); however, none of the biochemical indices measured in the blood sera showed significant effects of the treatments. According to Mazurkiewicz [22], all results of biochemical indices, except for the levels of ALT in the DN and Placebo groups, were within the reference range.

**Table 3:** Mean number of total white blood cells shown as a % of all counted cells in embryo blood samples from 20-day-old embryos after *in ovo* injection of curcumin (Cur), diamond nanoparticles (DN) and their bio-complexes 3Cur:1DN and 1Cur:3DN.

	Reference values	Group						ANOVA	
		Control	Placebo	Cur	DN	3Cur: 1DN	1Cur: 3DN	SEM	*P*-Value
**% WBC of total cell count**		0.7^a^	0.9^a^	2.6^b^	1.7^a^	1.1^a^	1.5^a^	0.517	0.0007
**AST [U/L]**	90–226	71.25	94.24	86.21	90.01	80.09	78.35	18.365	NS
**ALT [U/L]**	9–14	10.25	11.75	11.75	18.32	15.32	14.95	4.253	NS

ab values within rows with different superscripts are significantly different

#### Brain and liver histology

**Fig 11:**
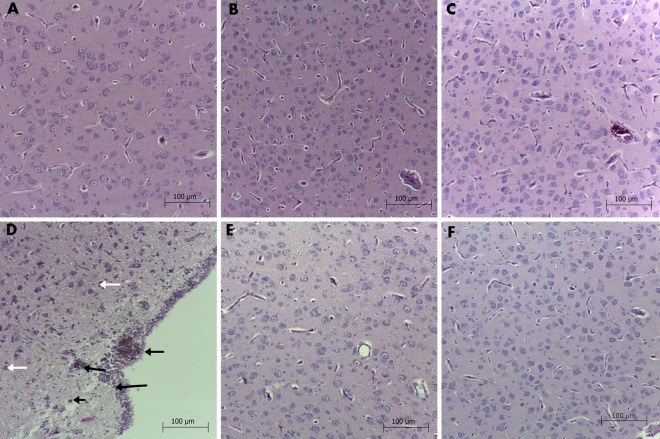
Brain histology of chicken embryo. **Groups: Control (A), Placebo (B), Cur (C), DN (D), 3Cur:1DN (E), 1Cur:3DN (F). Silver-grey particles were observed in the DN group (black arrows) and single necrotic-like neurons (white arrows).** Scale bar indicates 100 μm.

There were no noticeable changes or pathological features of brain microstructures in the inner part of the cerebral cortex in any of the groups. The average number of neural cells (area counted: 3,500 μm^2^) were not significantly different between the groups (Table 4). However, in the DN group, the outermost part of neuropilin was slightly thinner than in the control group (Fig 11) and some necrotic-like neurons were present. Silver-gray, round-shaped particles were observed in the outer part of the brain (Fig 11D).

**Table 4:** Mean number of neural cells counted in a total area of 3,500 μm^2^ in 20-day-old chicken embryos after *in ovo* injection of curcumin (Cur), diamond nanoparticles (DN) and their bio-complexes 3Cur: 1DN and 1Cur: 3DN.

	Group						ANOVA	
	Control	Placebo	Cur	DN	3Cur: 1DN	1Cur: 3DN	SEM	*P*-Value
**Number of neural cells**	471	506	511	487	495	521	14.2	NS

**Fig 12:**
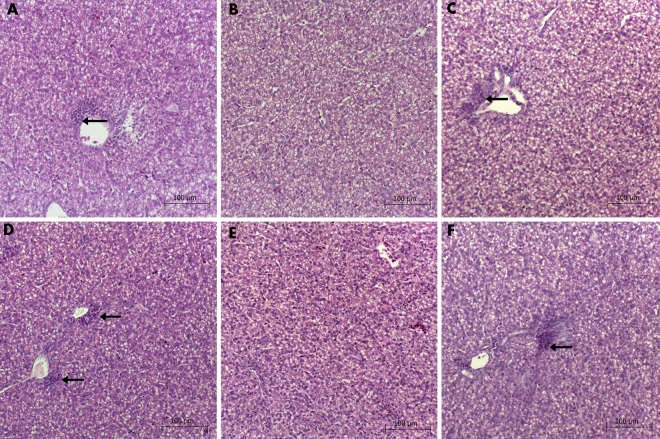
Liver histology of chicken embryos. **Groups: Control (A), Placebo (B), Cur (C), DN (D), 3Cur:1DN (E), 1Cur:3DN (F). Heterophils and primary hematopoietic cells were present in the proximity of wider blood vesicles (black arrows).** Scale bar indicates 100 μm.

No differences in liver histology were observed between the groups. In all groups, the abundance of fat droplets in hepatocytes was noticeable, which was due to glycogen accumulation since the 7th day of embryogenesis. In all groups, small populations of heterophils were present in the proximity of wider blood vessels and hepatic sinuses (Fig 12). This feature is not regarded as pathological, because of its presence in both Placebo and Control groups, at the same level as in the other groups. Along with the heterophils, darker and thicker cells are present, which were recognized as primary hematopoietic cells. In chicken, hematopoietic cells are moving toward the liver just before hatching and are usually abundant around the sinuses during this period.

## Discussion

In our experiment, we investigated the influence of Cur, DN and their bio-complexes on *in vitro* cell cultures and a chicken embryo model. By employing the cancerous cell line HepG2 and primary cell culture of normal fibroblasts, we expected differing responses to treatment with Cur and DN.

Cur has been used widely for centuries as a spice and is known to be non-toxic to humans and animals. It has been investigated mainly as an anti-inflammatory and chemopreventive agent, and numerous molecular targets have been found. Among them are genes involved in programmed cell death pathways. Thus, Cur has an enormous potential to eliminate cancer cells *in vitro* [3]. However, the application in *in vivo* systems remains the great challenge, as the bioavailability of Cur is poor, owing to its strong hydrophobic character [9].

In the present work, we show that Cur induces apoptotic death in cancer cells, but not in normal fibroblasts (Table 1; Figs 6 and 7). The proapoptotic effect of Cur against HepG2 cancer line was demonstrated previously. It is known that FAS, one of the target genes, that participates in fatty acid metabolism and lipogenesis, is downregulated by Cur and its intracellular protein activity is also inhibited, leading to the death of liver cancer cells [23]. Another proposed mechanism is the promotion of superoxide production by depletion of glutathione and Bcl-2 downregulation [24]. It should be noted that in our studies, the IC50 for HepG2 cells was calculated to be between 25.5 and 31.9 mg/L, which might seem relatively high to some authors [23,25]. However, most of the studies are based on solubilisation of Cur in organic solvents, such as DMSO, and then dilution in culturing media. Employing such chemicals for *in vivo* application might be controversial. Since the aim of the experiment was to increase the bioavailability of Cur in a hydrophilic environment, we did not use any solvents–Cur was only well dispersed in water by sonication.

Making a complex of Cur and a nanocarrier may improve Cur activity in a living organism. In the present experiment, we obtained bio-complexes of DN and Cur, which were proven to be stable, using Zeta potential measurement and TEM examination (Fig 1). DN can serve as a potential carrier of bioactive compounds such as curcumin. DN have a large surface area and a high absorption capacity [16], therefore, they can create bio-complexes with organic molecules such as L-glutamine [17]. Being one of the carbon allotropes, they are considered non-toxic and may be easily taken up by cells.

This was confirmed by the set of tests performed on *in vitro* cells of the HepG2 line and fibroblast culture, where the activity of Cur was increased when in a complex with DN. In the bio-complexes with DN, Cur showed higher activity than Cur itself, even in higher doses, e.g., in a Cur15:DN45 complex we observed lower viability of cells (Fig 2) and higher leakage of LDH (Fig 4) than in cells treated with Cur at 25‒75 mg/L concentrations. There was also the loss of proper morphology (Fig 8) and enhanced phosphatidylserine externalization (Fig 7),which is a feature of programmed cell death. Wang et al. [25] reported that loss of proper morphology after Cur treatment in this cell line is caused by the disruption of mitochondrial membrane potential and the disturbance of intracellular Ca^2+^ concentration. At the same time, HepG2 cells remained insensitive to bare DN (Figs 2, 4 and 6), as previously shown in other hepatic cancer cell lines [26], whereas, in fibroblasts, only a small effect was observed (Figs 3, 5 and 7). Interestingly, the assembly of Cur and DN seemed to have a negative impact on fibroblasts, indicating the interaction between Cur and DN (Table 1; Figs 5 and 7). Lundvig et al. [27] performed experiments on a similar primary culture of fibroblasts, showing that Cur indeed induced the apoptotic response by increasing reactive oxygen species production. Moreover, Zhou et al. [28] proved that Cur inhibited the proliferation of normal fibroblasts, not only *in vitro* but also *in vivo* in a mouse model of renal fibrosis, suggesting a PPARγ-dependent mechanism. The same receptor was recognized as a key molecule in angiogenic signal transduction in hepatic stellate cells [29], where Cur inducing its expression prevents hepatic fibrogenesis *in vitro* [30].

In the next step of the experiment, we decided to examine Cur, DN and their complexes on a living, tissue-organized organism. The investigation was carried out on the chicken embryo, which is a recognized animal model used in biocompatibility and toxicology trials and developmental studies employing nanoparticles and nanostructures [17,31–33]. Because it has a rapid growth rate and its development is well described, the model is relatively fast and reproducible. In our previous experiments, we have demonstrated that DN did not cause acute toxicity or increase mortality or affect the basic parameters of growth and development of embryos [17]. However, it was shown that DN decrease heart mass and reduce the number of blood vessels in both the developing chorioallantoic membrane of chicken and in glioblastoma multiforme tumour in *in ovo* experiments [19,20]. Moreover, DN were proven to inhibit the expression of bFGF, the factor controlling heart development [18]. A similar effect was observed in the present experiment: after treatment with DN and DN:Cur, the weight of the embryonic heart was reduced, compared with that of the control group (Table 2). It has been investigated that other carbon nanomaterials can also affect the microvasculature development, including microwave-radiofrequency carbon allotrope [20] or multiwalled carbon nanotubes, which decreased the formation of new vessels, while fullerenes C60 or graphene nanosheets were promoting the process [19]. Ema et al. [34] also reported the negative impact of both single- and multiwalled carbon nanotubes on foetuses of nanotubes treated mice, including malformation of the vasculature in placentas. Similarly, Qi et. al [35] described reduced number of blood vessels in murine placentas after 99^m^Tc-labelled oxidised multiwalled carbon nanotubes treatment in pregnant mice. Another carbon nanomaterial, pristine graphene did not affect the development of the embryos or their hearts in the experiment on chicken embryos, although it increased the mortality ratio [33].

Furthermore, the treatments caused a deposition of unidentified particles in the outer layer of the cerebral cortex (Fig 11). Round-shaped particles observed in the outer part of brains in the DN group could be aggregates, but they were observed only in the outermost layer of the brain tissue, beyond the ependyma (Fig 11D).

Yuan et al. [36] reported residues of DN in mouse bodies after a single intravenous injection. DN accumulated mostly in the liver and at a lower rate in the lungs. Very low uptake was noted in other tissues, including the brain. After a longer exposure (28 days) the level of accumulated DN was stable both in the liver and the lungs. In the cited work, the size of a single particle was approximately 50 nm, whereas the DN used in our studies ranged from 2 to 9 nm. It is known that the size of nanoparticles can influence their properties; therefore, comparisons between studies should be made carefully. Zhang et al. [37] employed DN of similar size (2 to 10 nm), radiolabelled with rhenium, and investigated their biodistribution and toxicity to mice after intratracheal instillation. They reported an accumulation of DN, mainly in the lungs and a redistribution in the other organs (spleen, liver and bones). Histology of the organs showed no abnormalities; however, histopathological analysis of lung tissues revealed inflammatory responses characterized by the accumulation of neutrophils and macrophages. In our experiment, the outer layer of neuropilin in the DN group was thinner than in the control group and some neuron cells showed features of necrosis: their nuclei were smeared and the cytoplasm was eosinophilic stained. However, the changes were not abundant, and the inner part of the brain was not different from that of the other groups. The formation of deposits in the brain may represent DN accumulation at the beginning of embryogenesis because the neural tube is the first structure to be developed [21].

In our previous works, we investigated the biocompatibility of DN in Wistar rats [15], knowing that DN can form large agglomerates in tissues [14]. Even though DN were administrated for four weeks and the agglomerates were present within a body cavity for three months, we noticed no major negative impact on the animals' health, neither did we observe a disruption of the oxidative state of the liver tissue nor a potential inflammatory state. Similarly, in the current experiment, we did not observe any signs of DN or DN:Cur accumulation in the chicken embryo liver. No pathological changes were observed in the liver histology (Fig 12).

Not many studies have been performed concerning embryotoxicity of Cur. Studies on the reproduction of Wistar rats supplemented with Cur showed no negative effects, neither on parental animals nor on the offspring [38]. On the other hand, Wu et al. [39] performed studies on zebrafish embryos and larvae and their results suggest that Cur negatively affects embryogenesis, causing numerous defects. Similarly, Chen et al. [40] reported that curcumin treatment caused apoptosis induction in mouse blastocysts *in vitro*. On the contrary, our results showed no negative effect on chicken embryo development.

The bio-complexes of DN and Cur did not affect embryo development, suggesting that the DN and Cur complexes may have different properties from those of raw compounds. It is possible that DN and Cur create a new compound, the properties of which are not a simple sum of the properties of DN and Cur. Interestingly, no deposits were observed in the brains, where DN were in a complex with Cur (Fig 11E–11F), which could indicate a specific interaction between DN and Cur and their active surfaces. On its own, DN tends to clump together due to its high surface energy [41]. Cur may attach to the reactive surface of DN, inhibiting the formation of the deposits during embryogenesis. All of the results from the DN and Cur groups were closer to those of the control group than to those of the DN group, confirming that the interaction between the compounds favours embryo development and that the bio-complexes are biocompatible. These observations support an argument for employing DN as a carrier for Cur or other active substances, which could prevent DN from clumping together, helping to disperse the particles properly in solutions, i.e., during the application of a drug based on DN. Complexes containing DN can be employed as a carrier for site-targeted therapies such as tumour treatment, promoting the accumulation and slow release of an active agent such as Cur in a tissue.

In our experiment, Cur was proven to induce apoptosis in liver cancer cells, which was significantly increased when Cur was in a complex with DN, indicating improved bioavailability of Cur. Even though such bio-complexes slightly affected normal cells *in vitro*, the effect was different from that seen in cancer cells and no harmful effects on the chicken embryo model were recorded. This favours the present bio-complex to be employed for liver cancer therapies, ensuring selective action against cancer cells without systemic harmful effects on normal tissues. The use of DN also has another benefit: DN itself may decrease the development of tumours, due to their antiangiogenic properties, which were investigated previously [18,19].

## Conclusions

*In vitro* experiments on HepG2 liver cancer cells confirmed the proapoptotic activity of Cur in cancer cells, but not in normal fibroblasts. The effect was significantly enhanced when Cur was in complexes with DN, indicating an improvement of Cur bioavailability in a hydrophilic environment. *In ovo* administration of a high concentration of hydrocolloids of DN, Cur and bio-complexes did not affect the development of chicken embryos, confirming their biocompatibility and selective Cur activity. Bio-complexes of DN and Cur are non-toxic, stable and biocompatible, which suggests their potential applicability in a drug delivery system such as cancer treatment without harmful effect on healthy tissues.
